# Sleep duration and food insecurity among minority urban college students: predictors of health and academic disparity

**DOI:** 10.3389/frsle.2026.1759416

**Published:** 2026-04-07

**Authors:** William Suarez-Gomez, Peter C. Nwakeze, Aditi Puri, Chesley Sanchez, Latoya Callender, Collette M. Brown

**Affiliations:** 1 Health, Equity, Administration and Technology Department, School of Health Sciences, Health Services and Nursing, Lehman College at the City University of New York, New York, NY, United States; 2 School of Allied Health Professions, King Graduate School, Monroe University, Bronx, NY, United States; 3 Department of Epidemiology and Biostatistics, School of Public Health, SUNY Downstate Health Science University, Brooklyn, NY, United States

**Keywords:** BMI, food insecurity, GPA, minority college students, sleep duration, sleep health

## Abstract

**Introduction:**

Synthesizing the literature on college students in urban settings, this study addresses how systemic disparities amplify food insecurity and poor sleep health in minority college students in the Bronx. We posit that their relationship is a predictor of higher body mass index (BMI) and lower grade point average to assess academic performance (GPA).

**Methods:**

This cross-sectional study investigated the association between self-reported sleep duration and food insecurity among 710 minority undergraduate students at two urban institutions in the Bronx, NY. The research used a QR-code-based survey to collect socio-demographics, food security data (US Household Food Security Module), and sleep duration (a PSQI sub-section). SPSS Version 29 was used for analysis. Multiple logistic regression was performed to examine the relationships between food security, sleep duration, demographics, BMI, and the GPA.

**Results:**

The findings reveal a high prevalence of food insecurity (52.1%) in this population. Chi-square analysis demonstrated statistically significant associations between food insecurity and household income (*p* < 0.0001), sleep duration (*p* = 0.007), and BMI (*p* = 0.037). A multiple logistic regression confirmed that obtaining the recommended sleep duration (7–9 h) was associated with 2.3 times higher odds of being food secure (*p* = 0.005, Exp(B) = 2.327). Additionally, students with a normal or overweight BMI were significantly more food secure than their obese counterparts.

**Conclusion:**

The current study highlights the interrelationship between sleep duration, food insecurity, socioeconomic status, and BMI among minority students. No correlation was observed between sleep duration, food insecurity, and GPA. However, our findings underscore the necessity for comprehensive, multifaceted interventions to effectively address these challenges faced by urban minority college students.

## 1 Introduction

The transition to higher education often brings significant financial burdens, compelling students to make difficult choices regarding basic needs like food and housing (Dickinson, 2023; El Zein et al., 2019; Powers, 2024). Adequate sleep is crucial for academic success and overall health, impacting cognitive functions and emotional regulation (Becerra et al., 2020; Hershner and Chervin, 2014; Hirshkowitz et al., 2015; Powers, 2024). Conversely, poor sleep is linked to chronic illnesses, mental health issues, and diminished academic performance (Al Salmani et al., 2020; Bermudez et al., 2022; Osei Bonsu et al., 2023; Zhou et al., 2022).

Food security, defined by the United States Department of Agriculture (United States Department of Agriculture Economic Research Service, 2025) as consistent access to adequate and safe food, is vital (El Zein et al., 2019; Sanborn et al., 2024). Food insecurity, on the other hand, is associated with various negative health outcomes, including chronic diseases, mental health problems like depression and anxiety, and impaired academic achievement (Austin and Smith, 2017; Bruening et al., 2018; Darling et al., 2017; Ding et al., 2015; Porras-Pérez et al., 2025; Rabbitt et al., 2023; Sharpe et al., 2016; Tseng et al., 2017).

These challenges are not evenly distributed. Food insecurity disproportionately affects minority racial and ethnic groups in the U.S., with non-Hispanic Black and Hispanic/Latinx households experiencing higher rates (Alhasan et al., 2023; Rabbitt et al., 2023). The Bronx in New York City (NYC) has the highest adult food insecurity rate (39%) among the boroughs (Chatterjee and Hinojosa, 2023; New York State Department of Health, 2023). Marginalized student groups, including African Americans, face heightened basic needs insecurity (Allen et al., 2018; Brown et al., 2025; Dong et al., 2023; Goldrick-Rab et al., 2019). For instance, the City University of New York, one of the largest university systems in the US, found that 48% of its undergraduates were food insecure, with Black and Hispanic students 1.5 times more likely to be affected (CUNY School of Public Health and Health Policy, 2020).

Sleep disparities frequently track with minority status and lower socioeconomic position, while suboptimal sleep durations remain a significant risk factor for obesity (Hale and Do, 2007; Frosztega et al., 2025; Whinnery et al., 2014). Black, Indigenous, and People of Color (BIPOC) students show significantly greater odds for both short and long sleep durations, indicating disrupted patterns (Bermudez et al., 2022; Alhasan et al., 2023). The rising costs of higher education intensify financial burdens, leading to increased food insecurity, which then cascades into detrimental impacts on sleep and mental health (El Zein et al., 2019; Powers, 2024). This pathway highlights how economic conditions profoundly influence student well-being. The existing racial and ethnic disparities in food insecurity and sleep health, combined with high local rates in the Bronx, amplify vulnerability for minority college students (Alhasan et al., 2023; Azhar et al., 2024; Bermudez et al., 2022; Goldrick-Rab et al., 2019; Hale and Do, 2007; New York State Department of Health, 2023; Rabbitt et al., 2023; Sanborn et al., 2024; Whinnery et al., 2014). Food insecurity might be used as a proximal determinant of sleep health, with affected individuals experiencing insomnia and/or short sleep duration compared to food-secure peers (Mazloomi et al., 2023). Short sleep duration is known to dysregulate appetite-signaling hormones which might exacerbate obesity trends within food-insecure populations. The concurrent effect of food insecurity and sleep duration necessitates investigation, particularly among urban college students. Currently, empirical work is lacking that simultaneously assesses the impact of food insecurity on sleep duration among college students while accounting for weight and academic performance. The theoretical Body Mass Index (BMI) is an acceptable benchmark to categorize underweight (<18.5), healthy weight (18.5–24.9), overweight (25.0–29.9), and obese (>30). The present study examined: how does the association between food insecurity and sleep duration relate to the BMI and academic performance (GPA) among minority students in urban colleges in the South Bronx? We posit that food insecurity and sleep duration are mutually influencing factors among minority students, leading to adverse outcomes. This group will demonstrate both higher BMI and lower GPA relative to their non-minority counterparts.

## 2 Literature review

This section systematically reviews the existing research on food security, sleep health, their interconnections, and the role of mental health, with a specific focus on college students and racial/ethnic disparities.

### 2.1 The landscape of food insecurity

Food security is formally categorized by the USDA into four levels: high, marginal, low, and very low (United States Department of Agriculture Economic Research Service, 2025). Conversely, food insecurity is defined as a household-level economic and social condition characterized by limited or uncertain access to adequate food, which is a distinction separate from individual hunger (Allen et al., 2018; Kendrick et al., 2022). This pervasive national challenge affected over 44 million people in the United States—including more than 13 million children—in 2022, underscoring its substantial and persistent scale (Allen et al., 2018; Alhasan et al., 2023; Rabbitt et al., 2023). However, addressing college food security effectively requires a systematic approach focused on identifying and closing priority research gaps to inform robust, scalable interventions (Landry et al., 2024).

#### 2.1.1 Food insecurity among college students

College students represent a particularly vulnerable demographic for food insecurity (FI). This susceptibility is largely attributed to limited financial resources, the diminishing purchasing power of federal aid, and the escalating costs of tuition, housing, and food (El Zein et al., 2019; Powers, 2024; Singh et al., 2024). The financial pressures of higher education frequently force students into precarious budgetary trade-offs, where limited funds must be partitioned between nutrition and essential costs like housing or textbooks (El Zein et al., 2019). Consequently, a significant portion of the US college students —estimated between 33% and 40%—shifts to half-time enrollment to accommodate full-time employment (Hanson, 2025; Leckrone, 2025). This transition often creates a policy paradox: while the income is necessary for survival, the employment and enrollment status can disqualify students from the Federal Supplemental Nutritional Assistance Program (SNAP), further exacerbating their food insecurity (USDA Food and Nutrition Service, 2025). The complexity of food insecurity (FI) among college students, often termed “hidden hunger,” highlights the need for a deeper understanding of their experiences (Fortin et al., 2020). A concept analysis identifies the core attributes of college student FI as insufficient food access, concurrent negative impacts on physical and psychosocial health and academic performance, and poor knowledge in navigating and utilizing available food resources (Hussain et al., 2022; Kendrick et al., 2022).

Studies conducted across the U.S. indicate a notably high prevalence of FI among university students. Rates have been reported to range widely from 12.5% to 84% in various studies, with a systematic review calculating an average prevalence of 42% among students (Bruening et al., 2018; Hussain et al., 2022). The prevalence and associated correlates of FI have been consistently documented across multi-campus university settings (Taylor et al., 2019). One multi-institutional study found that 19% of first-year college students were food-insecure, with an additional 25.3% identified as being at risk of food insecurity (El Zein et al., 2019). Food insecurity is significantly higher among U.S. college students (reporting a 40% reduction in diet quality or quantity) than the general population (10.2%) (Radtke et al., 2024). Rates exceeding 40% have been reported at various universities, including those on the East and West coasts of the US mainland (Adamovic et al., 2022; Brown et al., 2025; CUNY School of Public Health and Health Policy, 2020). For example, a study at a midsize rural university in Oregon found that 59% of students experienced food insecurity within the previous year, which was associated with fair/poor health, employment, and low income, and inversely with strong academic performance (Patton-López et al., 2014). This widespread occurrence confirms that student food insecurity is a systemic challenge embedded within the landscape of higher education, as further substantiated by scoping reviews (Nikolaus et al., 2020).

#### 2.1.2 Impact on academic performance

Food insecurity (FI) is demonstrably linked to detrimental effects on students' educational success and cognitive function (El Zein et al., 2019). Students experiencing FI frequently report a diminished ability to concentrate and consistently exhibit lower grade point averages (GPAs) (Bruening et al., 2018). This direct association highlights how the unmet basic need for adequate nutrition can undermine the fundamental mission of higher education. Beyond academic performance, FI has also been linked to poorer cognitive performance, cardiovascular disorders, and impaired executive functioning across the lifespan, with recent neuroimaging studies beginning to uncover the underlying neurological mechanisms (Guerithault et al., 2022; Porras-Pérez et al., 2025). Research on urban university undergraduate students further substantiates this, demonstrating a clear association between FI, academic performance, and related health behaviors (Ryan et al., 2022).

#### 2.1.3 Correlates with health and well-being

Among college students, FI is linked to poorer self-rated health and an increased risk of obesity (Allen, 2023; Knol et al., 2017). In chronic exposure, it is associated with serious long-term physical health issues, including cardiovascular disorders (Guerithault et al., 2022). The persistent and fluctuating nature of FI is also associated with adverse behavioral and mental health outcomes (Slotnick et al., 2023). Psychosocially, the experience of food insecurity can lead to profound psychological distress, including feelings of not being “worth food,” which negatively impacts students' overall well-being and academic engagement (Meza et al., 2019). The global relevance of this issue is underscored by comparative studies, such as one between Lebanon and Germany, which found a relationship between FI and lifestyle behaviors among university students (Rizk et al., 2023).

#### 2.1.4 Contributing factors and systemic links

Multidimensional factors converge to drive the elevated rates of FI observed among college students. Key contributors among diverse, urban college freshmen include severe financial constraints and the inherent challenges of transitioning to independent living (Bruening et al., 2016). Critically, food insecurity rarely occurs in isolation; it is deeply intertwined with other markers of instability. Studies demonstrate that FI is associated with housing instability, with both factors independently and synergistically compounding the negative impact on school performance among urban university students (Silva et al., 2017).

#### 2.1.5 Gender, racial, and ethnic disparities in food insecurity

Food insecurity disproportionately affects gender, racial and ethnic minority populations in the United States (Alhasan et al., 2023; Liu et al., 2014). This disparity is a critical aspect of understanding FI, as it reveals how systemic factors contribute to unequal burdens. Gender disparities in food security, dietary intake, and nutritional health are observed in women often facing a higher burden (Ma et al., 2021). Food insecurity in women consistently links to higher perceived stress and depressive symptoms, lower diet quality, and the most consistent observation of an association with obesity (Sharpe et al., 2016).

Pronounced disparities demonstrate that college students identifying as a racial minority are more likely to experience FI (Bruening et al., 2017; El Zein et al., 2019). Systemic and structural inequities can create and perpetuate disadvantages within these communities. For example, Black households consistently experience FI at rates more than double that of White non-Hispanic households, registering 21% and 28% compared to 8% and 13% respectively between 2016 to 2022 (Alhasan et al., 2023; Hales, 2024). Furthermore, a higher percentage of Black non-Hispanic (7.9%) and Hispanic/Latinx (5.1%) individuals appear to live in households with very low food security than White non-Hispanic participants (3.1%) (Allen et al., 2018; Ding et al., 2015).

Among Black non-Hispanic individuals, FI coupled with hunger shows a strong correlation with increased serious psychological distress. This mental health burden is critically compounded by the concept of “weathering,” which posits that the cumulative stress resulting from structural racism and discrimination contributes to an accelerated decline in health status among this population (Allen et al., 2018). Reflecting this impact on campus, FI is identified as a significant predictor of both depression and anxiety among the general college student population (Wattick et al., 2018). Consequently, effective interventions must move beyond immediate food provision to holistically address the underlying social determinants of health and structural racial injustice.

### 2.2 The landscape of sleep health

#### 2.2.1 Defining healthy sleep: duration and quality

Healthy sleep encompasses both adequate duration and good quality, both of which are essential for restorative properties and overall well-being (Hershner and Chervin, 2014; Jordan et al., 2016; Zhou et al., 2022). It is important to recognize that different ethnic and cultural minorities may have unique lifestyles, which can influence their sleep patterns as well (Lajunen et al., 2023). In the US, the National Sleep Foundation (2016) provides age-specific sleep duration recommendations, serving as guidelines for healthy individuals without underlying sleep disorders. Young adults aged 18 to 25 should aim for 7–9 h of sleep each night. It is 1 h longer than the new range proposed (6–8 h) for middle-aged adults based on intrinsic capacity (Chen et al., 2024). Sleeping significantly less or more than this suggested range may indicate underlying health issues or negatively impact a person's well-being (Fischer et al., 2021; Hirshkowitz et al., 2015; Jordan et al., 2016). There is also evidence that correlates unhealthy sleep patterns and shorter sleep duration with obesity occurrence (Alafif and Alruwaili, 2023; Li, 2021; Xu et al., 2025). Although the directionality between insufficient sleep and an increased risk of obesity seems unknown, sleep deprivation functions as a potent endocrine disruptor that destabilizes the metabolic environment; by suppressing leptin and elevating ghrelin, it alters energy homeostasis and significantly increases the risk of obesity (Broussard and Klein, 2022; Figorilli et al., 2025).

While self-reported sleep duration is a common metric, it is important to acknowledge that actual sleep time is often less than the total time spent in bed, which can introduce bias into self-reported data (Hirshkowitz et al., 2015; Jordan et al., 2016). Furthermore, assessing other critical aspects of sleep, such as sleep quality, sleep architecture (the cycling through different sleep stages), and the timing and regularity of sleep, is more challenging with self-reported measures. These elements are crucial contributors to the restorative benefits of sleep and require more objective measurement tools for a comprehensive understanding. The Pittsburgh Sleep Quality Index (PSQI) is a widely used self-report measure for sleep quality, and its psychometric properties have been evaluated in U.S. college students (Dietch et al., 2016; Zhou et al., 2022).

#### 2.2.2 Prevalence and consequences of sleep problems among college students

Daytime sleepiness, chronic sleep deprivation, and irregular sleep schedules are highly prevalent issues among college students, impacting a significant portion of this population (Hershner and Chervin, 2014; Zhou et al., 2022). A large multi-university study revealed that 62% of college students met the criteria for poor sleep, as indicated by a Pittsburgh Sleep Quality Index (PSQI) total score of 5 or higher. Within this group, 36% reported consistently obtaining less than 7 h of sleep per night (Becker et al., 2018). Students categorized as poor sleepers typically reported later bedtimes, long working hours, those employed in the service industry, experienced longer sleep onset latency (the time it takes to fall asleep), and obtained significantly less total sleep duration each night compared to their peers who reported good sleep (Becker et al., 2018; Chiang et al., 2020; Lu et al., 2024).

Several factors contribute to these widespread sleep problems. Physiologically, adolescents and young adults naturally exhibit a delayed circadian preference, often referred to as being “night owls,” which makes it difficult for them to fall asleep early enough to get sufficient sleep during the academic week (Hershner and Chervin, 2014). Emerging research highlights a gender-specific sleep gap proposing that women experience higher rates of insomnia driven by hormonal and social stressors, while men face frequent OSA underdiagnosis due to sex-divergent symptoms (Meira e Cruz and Andersen, 2025). Inadequate sleep hygiene, including irregular sleep-wake schedules, bedtime procrastination, noisy sleep environments, and the consumption of stimulants like caffeine after lunch or engaging in stimulating activities before bed, also plays a role (Al-Khalil et al., 2024; Hill et al., 2022). Alcohol use is associated with sleep patterns in first-year college students (Van Reen et al., 2016); specifically, the prevalent use of alcoholic beverages can initially induce sleep but subsequently leads to fragmented sleep (Hershner and Chervin, 2014). Energy drinks and binge drinking also predict college students' sleep quantity, quality, and tiredness (Patrick et al., 2016). Daily tobacco use has also been identified as a significant impediment to sleep among college students, associated with more sleep problems than binge drinking or illegal drug use (Boehm et al., 2016). Similarly, heavy use of technology, such as cell phones and computers, before bedtime exposes students to light that suppresses melatonin, further delaying sleep onset (Lastella et al., 2020). Furthermore, Gaultney (2010) found that 27% of college student participants were at risk for clinical sleep disorders (e.g., insomnia, restless legs, circadian rhythm disorders), directly resulting in increased sleepiness. Psychosocial correlates of insomnia among college students include stress, anxiety, and depression (Mbous et al., 2022). Whether caused by depression-induced bruxism or temporomandibular disorders or the pain of tissue damage, these emotional and/or sensory stressors significantly degrade sleep health (Seweryn et al., 2023; Wieckiewicz and Winocur, 2023). Sleep and eating disorders (anorexia, bulimia) and/or restriction of quantity and quality of foods that a person would need to keep healthy, and hunger can negatively affect sleep quality (Degasperi et al., 2024). Hedonic hunger, the desire to eat for pleasure rather than physiological need, has also been linked to subjectively assessed sleep quality and perceived stress among university students (Abdullaa et al., 2023). Recent studies indicate that sexual activity (partnered or solo) significantly influences sleep quality, specifically by increasing total sleep duration and reducing “wake after sleep onset (WASO) **(**Lastella et al., 2025). Marital satisfaction and co-sleeping dynamics also play a significant role in sleep quality (Andersen et al., 2025).

The consequences of poor sleep health among college students are severe, spanning both academic and physiological domains. Sleep deprivation fundamentally impairs cognitive function, learning, and memory consolidation, leading directly to lower grade point averages (GPAs) and an elevated risk of academic failure (Al Salmani et al., 2020). Notably, deviations from optimal sleep—including both short (<7 h) and long (>9 h) duration—independently predict worse academic performance during the first year of college, suggesting that dysregulation, not just deficit, is detrimental (Bermudez et al., 2022). Irregular sleep patterns, particularly late bedtimes, are consistently associated with reduced GPAs, while poor sleep more broadly impairs mood, increases depressive symptoms, and may elevate the risk of motor vehicle accidents due to drowsy driving (Hershner and Chervin, 2014). Moreover, pathological sleep patterns may also implicate impaired reproductive function and poor reproductive outcomes (Beroukhim et al., 2022; Cheng et al., 2023).

#### 2.2.3 Racial and ethnic disparities in sleep health

Racial and ethnic minorities in the United States experience significant disparities in sleep health across various dimensions (Alhasan et al., 2023; Hale and Do, 2007; Liu et al., 2014; Whinnery et al., 2014). This pattern of unequal sleep outcomes is a consistent finding in the literature, revealing a deeper layer of health inequity. Racial and ethnic differences in sleep duration have been suggested, with short sleep duration reported among Black non-Hispanics, Hispanics, and Asian non-Hispanics compared to White individuals (Alhasan et al., 2023).

Racial and ethnic disparities are evident in sleep duration, as Black/African American individuals are significantly more likely than White individuals to report very short (less than 5 h) and short sleep (5–6 h), with other Hispanics/Latinos and Asians/Others also demonstrating a higher likelihood of reporting very short sleep durations (Whinnery et al., 2014). Furthermore, Black respondents have an increased risk of being both short sleepers (≤ 6 h) and long sleepers (≥9 h) compared to White respondents (Hale and Do, 2007). This pattern of both short and long sleep being more prevalent among Black individuals suggests a broader dysregulation of sleep patterns, rather than just a simple lack of sleep. Temporal trends in racial and ethnic disparities in sleep duration among US adults from 2004–2018 show persistent differences (Caraballo et al., 2022).

Sleep disparities are evident across individual racial/ethnic subgroups within college student populations, including Black or African American, Hispanic or Latinx, Asian or Pacific Islander, and Multiracial students. Bermudez and colleagues (2022) mentioned that while multidimensional factors such as financial burden, employment status, and academic major explain some of the observed differences in sleep duration, these factors only partially account for the persistent disparities among BIPOC students. This persistence of disparities, even after controlling for common socioeconomic variables, strongly suggests that other unmeasured factors contribute significantly to these sleep differences. Anticipated and experienced discrimination, for instance, are identified as particularly relevant chronic stressors for BIPOC students, especially at predominantly White institutions, and are directly linked to poorer sleep outcomes (Bermudez et al., 2022). Poor sleep health among college students has been significantly associated with everyday discrimination (Becerra et al., 2020). Johnson et al. (2018) suggest that environmental determinants also play a role in insufficient sleep and sleep disorders, with implications for population health. Sleep patterns might be observed by race/ethnicity, serving as a marker of relative advantage or disadvantage (Johnson et al., 2019). Sleep health and serious psychological distress also show disparities across White, Black, and Hispanic/Latinx adults (Goldstein et al., 2020).

Racial/ethnic disparities in sleep health and potential interventions among women in the United States have also been explored (Jackson et al., 2020a). The causes and consequences of sleep health disparities are a significant area of research (Jackson et al., 2020b). Marital status is also associated with sleep health among Hispanics/Latinos in the US (Kim et al., 2021; Martinez-Miller et al., 2025). Cultural norms may also play a role in sleep disparities; for instance, Mexican immigrant men have been found to be significantly less likely than U.S.-born Mexican-Americans and other U.S. adults to report short sleep duration and insomnia, suggesting that Mexican cultural norms may be protective against insufficient and poor quality sleep (Seicean et al., 2011). This understanding implies that interventions aimed at improving sleep health for minority students must consider the profound and pervasive impacts of systemic racism and chronic stress, moving beyond individual-level behavioral changes to address broader social and environmental determinants of health.

### 2.3 The interplay of food insecurity and sleep health

#### 2.3.1 Direct associations between food insecurity and sleep outcomes (duration, quality, disturbances)

Food insecurity is consistently and robustly associated with adverse sleep outcomes across various dimensions in adults (Ding et al., 2015; Mazloomi et al., 2023; Osei Bonsu et al., 2023). These associations are not limited to specific age groups, or high-income countries, as food insecurity has also been found to be adversely associated with sleep duration, quality, and disturbances in older adults from low- and middle-income countries (Arzhang et al., 2024; Jacob et al., 2023). For instance, the risk of food insecurity and associated low sleep quality among pregnant women in the US is highest among 69% of the emerging adults and 57% in the young adults (Bailey et al., 2023). Beyond direct sleep outcomes, food insecurity is also linked to a range of other health issues, including poorer diet quality, increased risk of obesity, eating disorders, cardiometabolic diseases, stress, depression, and anxiety (Cheng et al., 2023; Guerithault et al., 2022; Phillips et al., 2024). A comprehensive systematic review and meta-analysis found that food insecurity significantly increased the risk of poor sleep quality (Odds Ratio = 1.45), shorter sleep duration (OR = 1.14), and longer sleep duration (OR = 1.14). It was also associated with an increased risk of specific sleep disturbances, including trouble falling asleep (OR = 1.39) and trouble staying asleep (OR = 1.91) (Ding et al., 2015; Mazloomi et al., 2023). The association with both shorter and longer sleep durations is particularly noteworthy, as it suggests a dysregulation of sleep patterns rather than a simple deficit, potentially reflecting deeper physiological or psychological stress responses to chronic food insecurity.

More specifically, very low food security has been linked to both very short sleep (OR = 1.86) and short sleep (OR = 1.44) (Ding et al., 2015; Whinnery et al., 2014). Among college students, food insecurity is consistently associated with worse sleep quality (Betancourt-Núñez et al., 2024; El Zein et al., 2019; Hagedorn et al., 2021; Radtke et al., 2024; Villela-Maciel et al., 2023). Food-insecure students, for instance, exhibited higher Pittsburgh Sleep Quality Index (PSQI) scores, a direct indicator of poorer sleep quality (Hagedorn et al., 2021; Villela-Maciel et al., 2023). Food insecurity is associated with poorer mental health and sleep outcomes in young adults (Nagata et al., 2019). The relationship between food insecurity and poor sleep health is complex and requires further disentanglement (Lee et al., 2021). Recent research specifically investigates the linkages between food insecurity, psychological distress, and poor sleep outcomes among U.S. college students (Arenas et al., 2019; Kopels et al., 2024). The association between food insecurity and insomnia symptoms among young adults in Puerto Rico also highlights the mediating role of psychological distress (Vazquez-Colon et al., 2024). Poor sleep health among college students has been significantly associated with food insecurity (Becerra et al., 2020).

Beyond self-reported measures, studies have corroborated these findings, with some using objective assessments of sleep. Troxel et al. (2020) suggest that greater food insecurity has been associated with objectively measured sleep problems, including shorter actigraphy-assessed sleep duration, poorer sleep efficiency, and poorer subjective sleep quality. They also said that food insecurity was linked to a greater likelihood of experiencing both short sleep (<7 h) and long sleep (>9 h) when measured objectively (Troxel et al., 2020). This reinforces the idea that food insecurity contributes to a broader disruption of healthy sleep patterns. Food insecurity has also been negatively correlated with sleep regularity, and this relationship can be mediated by dietary intake and serum concentrations of specific nutrients, underscoring the role of nutrition security (Degenhard et al., 2025). This aligns with broader findings that food insecurity is robustly associated with adverse sleep outcomes across various dimensions in adults (Ding et al., 2015; Mazloomi et al., 2023), and specifically with poor sleep health among college students (Becerra et al., 2020).

#### 2.3.2 Psychological distress as a mediator in the food insecurity-sleep link

Psychological distress consistently emerges as a significant pathway through which food insecurity influences sleep outcomes (Bermúdez-Millán et al., 2016; Mazloomi et al., 2023; Troxel et al., 2020). While the stress and anxiety associated with food insecurity can directly disrupt sleep, it is important to note that frequent mental distress only partially mediates the relationship between food insecurity and insufficient sleep (Liu et al., 2014). This indicates that while mental distress explains a considerable portion of the connection, food insecurity still independently contributes to insufficient sleep, suggesting that other factors, such as nutritional deficiencies or direct environmental stressors, also play a role (Nyer et al., 2013). However, the mental health dimension was not considered in the present study.

## 3 Materials and methods

### 3.1 Study design, setting, and participants

The study employed a cross-sectional design to examine the relationship between sleep duration and food security status among undergraduate students in the Bronx, NY. Participants were enrolled in private and in public urban colleges from January to May 2024, with the majority identifying as Hispanic or Black. The researchers used convenience sampling. Eligibility criteria for participation include being an undergraduate student aged 18 years or older.

The study included 710 undergraduate students. Before initiating data collection, the researchers obtained the necessary institutional permissions to access classrooms and to recruit participants from the selected urban colleges. Data were gathered through in-person and online methods. In-person data collection took place in classrooms. For online classes, researchers received Zoom links from several professors, allowing us to join the classes. Upon entering, researchers presented the study's objectives and invited students to voluntarily participate. Those professors who granted us access to their sessions were not involved in tracking participants. The students received no financial compensation or academic credit for submitting. Data were collected using a self-administered electronic survey accessed via a QR code.

### 3.2 Tool/questionnaires

The study employed a three-part, self-assessment digital questionnaire as the primary data collection instrument. This questionnaire was designed for efficient completion, with an estimated administration time of 20 min. The measures and associated covariates utilized within the instrument were collected via previously validated and reliable instruments, ensuring a robust foundation for subsequent statistical analysis.

#### 3.2.1 Demographic variables

The study utilized a comprehensive set of demographic and contextual variables, assessed using categorical options, to characterize the sample. These included race and ethnicity (based on self-identification), level of education, income, age, gender, household size, and number of children, alongside self-reported health status, height, and weight. Anthropometric data, specifically the Body Mass Index (BMI), was estimated using the self-reported height and weight (weight divided by height squared, kg/m^2^). The Grade Point Average (GPA) was categorized into three distinct groups for analysis: below 2.5, 2.5–3.49, and 3.5–4.0. Furthermore, the study captured variables related to food assistance access, including participants' receipt of SNAP or WIC benefits, and their awareness and utilization of an on-campus food pantry.

#### 3.2.2 Sleep duration

The Pittsburgh Sleep Quality Index (PSQI) short version is a clinically validated, five-component, self-report tool for college students that typically assesses Sleep Quality over “the past month” (Radtke et al., 2024). It demonstrates strong internal reliability (Cronbach's α up to 0.83) and high specificity (86.5%) (Buysse et al., 1989). In our study, only the component assessing “sleep duration” was measured and used as the dependent variable, based on the following questions: “On average, about how many hours of sleep do you get per night?” “Do you have difficulty getting up in the morning?” ‘Do you fall asleep easily during the day?” “Do you have difficulty concentrating, being productive, and completing tasks at work or at school?” “Are you restless during sleep, tossing and turning from one side to another?” Responses to the questions included less than 5 h, 5–5.9 h, 6–6.9 h, and 7–9 h. Watson et al. (2015) recommend that adults regularly get 7 or more h of sleep per night to promote optimal health. Therefore, for the purpose of this study, sleep duration responses will be reported as less-than-recommended sleep (less than 5–6.9 h) or recommended sleep (7–9 h).

#### 3.2.3 Food security

Food insecurity (FI) was measured using the US Household Food Security Survey Module (ten-item questionnaire) developed by the Economic Research Service of the US Department of Agriculture (USDA Economic Research Service, 2012). The instrument has a Cronbach alpha of 0.856 (Hamilton et al., 1997). Participants were asked to recall how frequently they faced challenges in obtaining sufficient food over the previous year. Responses were categorized as “often true” or “sometimes true,” both of which were coded as affirmative and assigned a score of 1, while “never true” was assigned a score of 0. Responses of “Don't know” or “refused” were also scored as 0. The total score was calculated by summing the affirmative responses. Based on the USDA guidelines, survey is scored on a scale of 0 to 6, with a score of 0–1 indicating high or marginal food security, 2–4 indicating low food security, and a score of 5–6 indicating very low food security. For analysis, these scores were dichotomized: 0–2 categorized as food secure and 3–10 as food insecure.

### 3.3 Analysis

Data were analyzed using the Statistical Package for the Social Sciences (SPSS, v. 29) software (IBM Corp, 2025). Descriptive statistics and univariate analyses were calculated, followed by a Chi-square test to assess initial associations. Cross-tabulations were then performed using sleep duration and food security status (food secure and food insecure) and demographic covariates used in the model were age, race, ethnicity, education, income, age, gender, SNAP/WIC participation, household size, number of children in the household, self-reported health, BMI, and GPA. Multiple logistic regression was used to control for variables that could potentially skew the findings (*p* < 0.1). Log-odds [lnExp(B)] were used instead of odds ratios to directly compare the effect size and direction across models. Values of *p* < 0.05 indicated statistical significance.

### 3.4 Ethical considerations

Ethical approval for this study was obtained from the Institutional Review Board (IRB) in Lehman College at CUNY (IRB No. 2024-0087) and Monroe University (IRB No: FAC-2023-04), respectively, both in NY. Each survey included an informed consent form. Before starting the survey, the participants had to read and accept the informed consent form. Affirmative consent was obtained when students selected “yes” to the question, “Do you want to participate in this study?” Students were also invited to take a screenshot of the consent form for their own records. All participants provided informed consent, electronically, using the digital platform (Survey Monkey). To uphold privacy, security, and confidentiality, the data was devoid of any information that could potentially identify the participants, and IP addresses were not collected.

## 4 Results

### 4.1 Sample demographics

Table 1 describes the characteristics of undergraduate participants from two urban colleges in Bronx, New York. Seven hundred and ten students participated in the study. Most of the students were between 18 and 24 years old (67.1%), females (73.3%), Black non-Hispanic (44.4%) and Hispanic (41.5%), and more than half had a household income below $40,001 (58.3%). Regarding body mass index (BMI), 28% obese. Students from all academic undergraduate levels were included; most were freshmen (39.4%), and 60% reported an academic performance (GPA) between 2.5 and 3.49.

**Table 1:** Demographic characteristics of the participants.

Variables	Number	Percentage
Age (*n =* 709)		
18–24	476	67.1
25–34	141	19.9
>35	92	13.0
Gender (*n =* 704)		
Male	181	25.7
Female	523	73.3
Race/ethnicity (*n =* 703)		
AI/AN	10	1.4
Asian (non-Hispanic)	42	6.0
Black/African American	312	44.4
Hispanic	292	41.5
Native Hawaiian/Pacific	5	0.7
White (non-Hispanic)	42	6.0
Education (*n* = 710)		
Ass degree (1^st^ yr)	280	39.4
Ass degree (2^nd^ yr)	147	20.7
Bach degree (3^rd^ yr)	136	19.2
Bach degree (4th yr)	147	20.7
GPA (*n =* 657)		
>2.5	65	9.9
2.5–3.49	396	60.3
3.5–4.0	196	29.8
Income (*n =* 689)		
<$20,000	211	30.6
$20,000–$40,000	191	27.7
$40,001–$60,000	127	18.4
$60,001–$80,000	80	11.6
<80,000	80	11.6
Sleep duration (*n =* 705)		
<5 h	82	11.6
5.0–5.9	218	30.9
6.0–6.9	262	37.2
7.0–9.0	143	20.3
BMI (*n =* 676)		
Underweight	24	3.5
Normal weight	286	42.3
Overweight	177	26.2
Obese	189	28.0
Have children (*n =* 710)		
Yes	146	20.6
No	564	79.4
No. of children (*n =* 142)		
1–3	130	91.5
4–7	12	8.5
Food pantry (*n =* 706)		
Yes	248	34.3
No	107	14.8
Don't know	351	48.6
Received food from pantry (*n =* 247)		
Yes	104	41.9
No	143	57.7
Food security status (*n =* 710)		
Food secure	334	46.3
Food insecure	376	52.1

Only 20.6% of the students had children, with approximately 92% reporting having 1–3 children. Almost 42% of all participants sleep less than 6 h per day. The prevalence of food insecurity among students who attended these colleges was 52.1%. Almost half of the students (48.6%) did not know whether their college has a food pantry. Among those who knew whether their college has a food pantry, approximately 42% reported receiving food from the pantry.

Based on all respondents, a chi-squared test was performed to identify the relationship by sleep duration (Table 2). Among students who self-assessed less than 7 h of sleep a day, the distribution is as follows: 80.3% are freshmen, 78.8% are sophomores, 74.8% are juniors, and 83.7% are seniors. Approximately 46% show an obese BMI. Also, 82.9% of the students with 1 or more children reported sleeping less than 6.9 h per day. The data reveal a pattern in which lower income is associated with shorter sleep duration, particularly by ethnicity and BMI. For instance, Non-Hispanic White students were more likely to get 7–9 h of sleep per night compared to other groups (chi-square 37.62, df = 12, *p* < 0.001). Students who were obese were less likely to get 7–9 h of sleep per night (chi-square 19.87, df = 9, *p* = 0.019). No other variables in the bivariate analysis were statistically significant.

**Table 2:** Demographic variables and sleep duration.

Variables	Sleep duration (hours)					
	>5 h (%)	5–5.9 h (%)	6–6.9 (%)	7–9 h	df	*p*-value
Age						
18–24	53 (11.2)	130 (27.4)	186 (39.2)	105 (22.2)	6	0.069
25–34	14 (9.9)	56 (39.7)	47 (33.3)	24 (17.0)		
>35	15 (16.5)	31 (34.1)	30 (33.0)	15 (16.5)		
Gender						
Male	12 (6.7)	54 (30.3)	69 (38.8)	43 (24.2)	3	0.127
Female	65 (12.4)	164 (31.4)	194 (37.1)	100 (19.1)		
Race/ethnicity						
AIAN	3 (30.0)	1(10.0)	3 (30.0)	3 (30.0)	12	<0.001^*^
Asian (non-Hispanic)	4(9.5)	12 (28.6)	16 (38.1)	10 (23.8)		
Black (non-Hispanic)	37 (11.9)	116 (37.4)	118 (38.1)	39 (12.6)		
Hispanic	31 (10.7)	81 (27.8)	107 (36.8)	72 (24.7)		
White (non-Hispanic)	2 (4.8)	7 (16.7)	15 (35.7)	18 (42.9)		
Education						
Ass degree (1^st^ yr)	37 (13.2)	91 (32.5)	97 (34.6)	55 (19.6)	9	0.569
Ass degree (2^nd^ yr)	14 (9.6)	40 (27.4)	61 (41.8)	31 (21.2)		
Bach degree (3^rd^ yr)	16 (11.9)	37 (27.4)	48 (35.6)	34 (25.2)		
Bach degree (4th yr)	15 (10.2)	50 (34.0)	58 (39.5)	24 (16.3)		
GPA						
>2.5	11 (16.9)	23 (35.4)	20 (30.8)	11 (16.9)	6	0.621
2.5–3.49	43 (10.9)	115 (29.0)	155 (39.1)	83 (21.0)		
3.5–4.0	20 (10.4)	56 (29.0)	76 (39.4)	41 (21.2)		
Income (US$)						
<20,000	30 (14.3)	75 (35.7)	69 (32.9)	36 (17.1)	12	0.256
20,000–40,000	19 (9.9)	51 (26.7)	83 (43.5)	38 (19.9)		
40,001–60,000	16 (12.7)	34 (27.0)	50 (39.7)	26 (20.6)		
60,001–80,000	10 (12.5)	25 (31.3)	28 (35.0)	17 (21.3)		
<80,000	5 (6.3)	27 (34.2)	25 (31.6)	22 (27.8)		
Food security status						
Food insecurity	50 (15.1)	103 (31.0)	126 (38.0)	53 (16.0)	3	0.007^*^
Food security	32 (8.6)	115 (31.0)	136 (36.5)	90 (24.1)		
BMI						
Underweight	6 (25.0)	2(8.3)	6 (25.0)	10 (41.7)		
Normal weight	24 (8.4)	85 (29.7)	118 (41.3)	59 (20.6)		
Overweight	20 (11.4)	57 (32.4)	63 (35.7)	36 (20.5)		
Obese	24 (12.7)	65(34.4)	66 (34.9)	34 (18.0)	9	0.019^*^
Have children						
Yes	24 (16.7)	45 (31.3)	51(35.4)	24 (16.7)	3	0.146
No	58 (10.3)	173 (30.7)	212 (37.7)	120 (21.3)		
No. of children						
None	7(15.2)	12 (26.1)	15 (32.6)	12 (26.1)	6	0.823
1–3	21 (16.3)	38 (29.5)	48 (37.2)	22 (17.1)		
4–7	2 (16.7)	5 (41.7)	3 (25.0)	2 (16.7)		

^*^denotes statistically significant variables.

The data reveal an inverse pattern in which lower income is more likely associated with shorter sleep duration. For instance, the proportion of individuals sleeping less than 7 h decreases steadily as income rises, ranging from 82.9% for those earning under $20,000 down to 72% for those in the highest income bracket (above $80,000). Those who reported earning less than $20,000 had the highest percentages of people sleeping fewer than 5 h (14.3%) and 5–5.9 h (35.7%). Conversely, the highest income group (above $80,000) reported the lowest percentage of people sleeping less than 5 h (6.3%) and the highest percentage of people achieving sufficient sleep, defined as 7–9 h (27.8%). The middle-income group ($20,000-$80,000) had the largest percentage of individuals sleeping 6–6.9 h, suggesting this range is common but insufficient for many participants regardless of income. The observed distribution of percentages suggests an inverse relationship, where increasing income is positively associated with a greater likelihood of obtaining seven or more hours of sleep (*p* = 0.256). In the context of FI, students which household incomes above $60,000 were more food secure than those who reported lower incomes (Table 2).

Regarding the GPA, 52.3% of those below 2.5 reported sleeping fewer than 6 h per day, which is at least 13% higher than students with a GPA above 2.5. This value is at least 13% higher compared to students with a GPA above 2.5, but not statistically significant (*p* = 0.621).

Among those foods insecure only 334 completed the sleep-duration tool. Based on this cohort, the bivariate analysis (Table 3) reveals several statistically significant associations between food insecurity, income, sleep duration, and BMI. Specifically, income exhibits the strongest correlation, with students in the lowest annual income (<$20,000) bracket experiencing the highest food insecurity at a rate of 53.6%, which decreases markedly in higher income categories, confirming a robust link between economic vulnerability and unstable access to food (*p* < 0.001). Students who reported higher household incomes above $60,000 were more food secure (67.5%) than those who reported lower incomes.

**Table 3:** Participants (food insecure vs. secure) by demographic variables and sleep duration.

Variables	Food insecure *n* (%)	Food secure *n* (%)	DF	*P*
Age				
18–24	211 (44.3)	265 (55.7)	2	0.104
25–34	75 (53.2)	66 (46.8)		
>35	48 (52.2)	44 (47.8)		
Gender				
Male	85 (47.0)	96 (53.0)	1	0.978
Female	245 (46.8)	278 (53.2)		
Race/ethnicity				
AIAN	5 (50)	5 (50)	5	0.051
Asian (non-Hisp.)	18 (42.9)	24 (57.1)		
Black (non-Hisp)	163 (52.2)	149 (47.8)		
Hispanic	128 (43.8)	164 (56.2)		
White (non-Hisp)	12 (28.6)	30 (71.4)		
Acad. education				
Asso. degree (1st yr)	141 (50.4)	139 (49.6)	3	0.553
Asso. degree (2nd yr)	66 (44.9)	81 (55.1)		
Bach degree (3rd yr)	62 (45.6)	74 (54.4)		
Bach degree (4th yr)	65 (44.2)	82 (55.8)		
GPA				
>2.5	37 (56.9)	28 (43.1)	2	0.242
2.5–3.49	181 (54.7)	215 (54.3)		
3.5–4.0	94 (48.0)	102 (52.0)		
Income				
<20,000	113 (53.6)	98 (46.4)	4	<0.001^*^
20,000–40,000	92 (48.2)	99 (51.8)		
40,001–60,000	69 (54.3)	58 (45.7)		
60,001–80,000	26 (32.5)	54 (67.5)		
<80,000	25 (31.3)	55 (68.8)		
Sleep duration				
<5 h	50 (61.0)	32 (39.0)	3	0.007^*^
5.0–5.9	103 (47.2)	115 (52.8)		
6.0–6.9	126 (48.1)	136 (51.9)		
7.0–9.0	53 (37.1)	90 (72.9)		
BMI				
Underweight	10 (41.7)	14 (58.3)	3	0.037^*^
Normal weight	124 (43.4)	162 (56.6)		
Overweight	76 (42.9)	101 (57.1)		
Obese	105 (55.6)	84 (44.4)		
Have children				
Yes	78 (53.4)	68 (46.6)	1	0.083
No	256 (45.4)	308 (54.6)		
No. of children				
None	31 (67.4)	15 (32.6)	2	0.161
1–3	68 (52.3)	62 (47.7)		
4–7	8 (66.7)	4 (33.3)		

^*^denotes statistically significant variables.

A chi-square test was performed to determine the association between food security and the demographic variables and sleep duration. There was a statistically significant relationship between sleep and food insecurity (*p* = 0.007). Those who slept <5 h a day were most likely to be food insecure (61%). Similarly, students who were obese showed higher levels of food insecurity (55.6%) compared to those who had normal weight (43.4%).

### 4.2 Sleep and food insecurity

The logistic regression analysis (Table 4) examined the factors associated with the likelihood of obtaining sufficient sleep (defined as an average of seven or more hours per night). Results indicated that food security was positively associated with getting enough sleep [B = 0.411, *p* = 0.044, Exp(B) = 1.509, 95%CI: 1.011–2.252]. Students who were food secure were 1.5 times more likely to get the recommended hours of sleep per night (7–9 h). This indicates that individuals who are food secure have approximately 51% higher odds of achieving 7 or more h of sleep than those who are food insecure, holding all other variables constantly.

**Table 4:** Logistic model on effects of food insecurity and demographic variables on sleep duration.

Variables	B	S.E.	Wald	df	Sig.	Exp(B)	95% CI for Exp(B)	
							Lower	Upper
Food security	0.411	0.204	4.046	1	0.044^*^	1.509	1.011	2.252
Age 18–24	0.240	0.322	0.556	1	0.456	1.271	0.677	2.389
Age 25–34	−0.042	0.378	0.012	1	0.912	0.959	0.457	2.013
Non-Hispanic AI/AN	−1.294	0.918	1.985	1	0.159	0.274	0.045	1.659
Non-Hispanic Asian	−1.013	0.502	4.063	1	0.044^*^	0.363	0.136	0.972
Non-Hispanic Black/African American	−1.534	0.358	18.352	1	<0.001^*^	0.216	0.107	0.435
Hispanic	−0.658	0.340	3.746	1	0.053	0.518	0.266	1.008
Underweight	1.220	0.496	6.045	1	0.014^*^	3.386	1.281	8.953
Normal weight	−0.007	0.258	0.001	1	0.977	0.993	0.599	1.646
Overweight	0.094	0.278	0.115	1	0.735	1.099	0.637	1.894
Constant	−0.866	0.452	3.674	1	0.055	0.421		

^*^denotes statistically significant variables.

### 4.3 Sleep and racial/ethnicity variables

The most pronounced relationships were observed among the racial/ethnic variables, with both Asian Non-Hispanic and Black Non-Hispanic groups showing significantly lower odds of achieving sufficient sleep compared to the reference group. Students who identified as Asian-Non-Hispanic [B = −1.013, *p* = 0.044, Exp(B) = 0.363, 95% CI: 0.136–0.972] and Black-Non-Hispanic [B = −1.534, *p* < 0.001, Exp(B) = 0.216, 95% CI: 0.107–0.435] were significantly less likely to report sleeping 7–9 h per night compared to the White-Non-Hispanic students. The Hispanic group also showed a strong but marginally significant trend toward lower odds [Exp(B) = 0.518, *p* = 0.053], reinforcing the pattern of minority groups facing sleep disadvantages. The Black Non-Hispanic group exhibited the strongest effect, with nearly 78% lower odds of sleeping 7 or more h.

### 4.4 Sleep and body mass index

A significant finding emerged concerning Body Mass Index (BMI) status, specifically for the Underweight category. Individuals classified as underweight had an Odds Ratio of 3.386 (*p* = 0.014), over two times more likely to report seven or more hours of sleep per night compared to those obese. This unexpected and substantial positive association suggests a complex link between being underweight and having higher odds of sufficient sleep, potentially related to underlying health conditions or lifestyle factors common in this group. In stark contrast, the Normal Weight and Overweight categories showed no statistically significant relationship with the likelihood of sufficient sleep.

## 5 Discussion

This cross-sectional study investigated the intricate interrelationships among food security status, sleep duration, BMI and GPA among 710 undergraduate students drawn from two urban colleges. Specifically, the research provides detailed insights into the prevalence of poor sleep duration within the food-insecure student minorities population residing in the South Bronx, New York. The comprehensive breakdown of the study participants reveals a student body that is not only ethnically diverse but also significantly financially constrained, providing a nuanced view of the population under investigation. Our findings about sleep health disparities across various dimensions experienced by racial and ethnic minorities align with existing literature (Alhasan et al., 2023; Hale and Do, 2007; Liu et al., 2014; Whinnery et al., 2014). Specifically, short sleep duration among food-insecure populations is more common in Black non-Hispanics, Hispanics, and non-Hispanic Asian individuals compared to White individuals (Alhasan et al., 2023).

The high percentage of freshmen (39.4%) and sophomores (20.7%) indicates a younger student population. The income distribution indicates that a significant majority of students fall within the extremely low to very low-income brackets relative to regional benchmarks. Specifically, 29.8% of students reported an annual income of less than $20,000, while 26.6% earned between $20,000 and $39,000. For comparison, the median income for the U.S. Northeast region was $92,000 in 2024 (US Census Bureau, 2025). Relative to the US national figures, these students remain within the lowest national quartile which applies to earning less than $34,510. This financial profile is a crucial context for understanding the high rate of food insecurity observed in the study. Regarding the income, the evidence provides a robust relationship (*p* < 0.001) that food insecurity is not randomly distributed across income levels. Table 2 quantifies the percentages of food-insecure and food-secure individuals within each variable category, providing a clearer picture of the disparities. The prevalence of food insecurity among undergraduate students demonstrates a significant inverse relationship with income. Specifically, students from the lowest income bracket (under $20,000) experienced a food insecurity rate of 53.6%, which decreased substantially to 31.3% among those in the highest bracket provided (over $80,000). This financial constraint is posited to influence adverse dietary coping mechanisms, including an increased frequency of meal skipping and a greater reliance on energy-dense, highly processed, and nutritionally deficient food options (e.g., high carbohydrate-loaded snacks) due to their lower cost.

This research revealed that minority students experiencing low income, normal and high **BMI**, and food insecurity are significantly associated with poor sleep quality. The relationship between BMI and food security is made more explicit; 55.6% of obese students were food insecure, a higher proportion than any other BMI category. Similarly, the Exp(B) values for BMI categories provide quantitative evidence for the relationship with food security. The Exp(B) of 1.529 for normal weight and 1.614 for overweight show that students in these categories are about 1.5 to 1.6 times more likely to be food secure than the reference group, which is likely the obese category (Table 3). The significant *p*-values for both normal (*p* = 0.028) and overweight (*p* = 0.025) categories provide further statistical backing for this finding. These regression results highlight that income, sleep, and BMI are not just correlated with food insecurity but are strong, independent predictors of a student's food security status. This study aligns with Dong et al. (2023), which found that food insecurity among native minorities in Alaska is linked to higher body mass index (BMI) and suggests that sleep health may mediate the negative effects on cardiometabolic risks.

The current study did not detect significant **gender** differences in sleep quality, contrasting with reports of higher sleep difficulties among women in other populations (Villela-Maciel et al., 2023). Existing literature consistently highlights that gender moderates food insecurity, making women—particularly those who are independent or caregivers—more susceptible to the combined burden of poor sleep and food insecurity (Osei Bonsu et al., 2023). Furthermore, specific studies show severe risks for pregnant women, linking food insecurity to low sleep quality and the adoption of obesogenic-promoting behaviors associated with increased stress and depression (Bailey et al., 2023; Cheng et al., 2023). While the present analysis did not account for pregnancy status, hormonal factors (e.g., menstrual cycles, menopause), or sleep aids, we found no significant interaction between sex, food insecurity, and sleep duration (*p* > 0.9).

Regarding the **sleep duration**, our study shows a significant majority (79.7%) do not get the recommended 7–9 h of sleep, with most reporting 5.0–6.9 h. This aligns with the overall literature on poor sleep among college students (Becker et al., 2018; Bermudez et al., 2022; Hershner and Chervin, 2014). Factors such as sleep habits, work hours, and parenting responsibilities may predict academic performance among student employees (Chiang et al., 2020). For students facing financial challenges, securing a job is particularly essential to cover tuition and living expenses. However, participants who reported sleeping 7–9 h a night experienced food security more frequently than those who slept for fewer hours. The multiple logistic regression analysis presented in Table 3 provides a clearer predictive insight into the data. The Exp(B) values, commonly referred to as odds ratios, represent the most critical information in this table. In terms of sleep duration, this variable reaffirms its statistical significance. The Exp(B) value of 2.327 for the 7–9 h sleep category suggests that, when other factors are held constant, students who rate themselves as food secure are 2.327 times more likely to obtain adequate sleep than their food-insecure peers (*p* < 0.005). This finding is in line with the research conducted by Jacob et al. (2023), which indicates that addressing food insecurity and its potential mediators could help mitigate sleep issues.

The observed lack of a statistically significant relationship between academic performance and the intersection of food insecurity and sleep duration was unexpected, diverging from established literature (Bermudez et al., 2022; Bruening et al., 2018; El Zein et al., 2019; Hershner and Chervin, 2014). We speculate that this non-significant finding may stem from several possibilities: the study's high proportion of freshmen participants, who reported GPA based on only one semester; the use of self-reported data, which prevented us from ascertaining participants' true GPA; and the potential for participants to provide socially desirable responses. On the other hands, students might employ temporary coping strategies that mitigate the effect of long term food and sleep issues on their performance (Mitchell et al., 2022); the effects of these influences may be harder to detect in young adults than in younger groups, due to varying levels of vulnerability or resilience between the two populations (Osei Bonsu et al., 2023); and/or the rise of synchronous online education may enhance access to resources during assessments, potentially masking the true cognitive load and its impact on academic outcomes compared to in-person settings (Hung et al., 2024).

Disparities in student welfare are evident through the confluence of environmental barriers and institutional resource gaps. Although precise residential data was not collected, participants residing in the Southern Bronx are generally impacted by “sleep deserts,” which are environments detrimental to optimal sleep health (Dunietz et al., 2022). However, these factors require contextual assessment. Furthermore, a significant lack of awareness regarding campus-based food resources was observed, with nearly half of the students reporting they were unaware of the available food pantry. This limited knowledge for navigating and utilizing food support aligns with external findings (Bruening et al., 2017; El Zein et al., 2019; Hussain et al., 2022; Kendrick et al., 2022), underscoring an important gap in institutional support, particularly given that only one of the referred campus locations (Lehman College) operates a food pantry program.

## 6 Implications

The combined evidence strongly suggests that food insecurity represents a significant precursor to stress among college students, which subsequently predisposes them to the development of unhealthy sleep patterns, thereby compounding the negative academic and health-related outcomes these students encounter (Bruening et al., 2018; El Zein et al., 2019; Hershner and Chervin, 2014; Al Salmani et al., 2020). The compounding effect of these multiple challenges establishes a significant barrier to student success, considering that poor sleep quality—encompassing issues with duration, continuity, and overall satisfaction—is linked both independently and interactively with increased psychosocial stress, ultimately diminishing mental well-being, academic performance, and productivity. Instead of normalizing inadequate sleep as a requisite academic sacrifice, institutions should actively promote “restorative sleep”, recognizing its fundamental role in student well-being, especially given that the pervasive use of technological devices poses an increasing challenge to maintaining healthy sleep hygiene (Robbins et al., 2022). Although the term “restorative sleep” lacks a universally accepted definition, it can be described as a complex physiological state marked by refreshment and alertness after a sufficient duration of consistent sleep. This experience is further enriched by positive cognitive and emotional facets of rest. To enhance sleep hygiene, the initial step is to cultivate awareness of its significance.

The variation in sleep and sleep habits across diverse countries and settings is a critical, yet underdeveloped area of study (Pilcher et al., 2024). Evidence suggests that differences in sleep duration may correlate with cultures, national happiness, moderate individualism, obesity, and schooling, particularly in highly industrialized nations (Lajunen et al., 2023). Consequently, multicultural institutions should implement culturally aware strategies to address sleep-related issues and food insecurity in educational settings (Ampofo et al., 2025). Furthermore, this intricate dynamic is compounded in vulnerable communities by the coexistence of “sleep deserts” and “food deserts.” The connection between these “deserts” highlights a crucial aspect of environmental inequality, which together may trigger a cascade effect of metabolic dysfunctions and disturbances, contributing to obesity, chronic health conditions, and health disparities among residents (Dunietz et al., 2022). In addition, aligning with literature linking multiple unmet needs (food insecurity, housing instability, poor mental health) to adverse academic outcomes like dropout (Sanborn et al., 2024). A holistic understanding of sleep regularity is essential, necessitating the parallel measurement of co-varying behaviors such as light exposure, meal timing and type, and college activities (Fischer et al., 2021). All these factors emphasizes the urgent need for developing and testing novel interventions that holistically target students with multiple unmet essential needs to contribute meaningfully to student retention and higher graduation rates (Sanborn et al., 2024).

While not directly assessed in this research, we recognize that the literature highlights several other factors related to “sleep health” that negatively impact academic outcomes and align with aspects of poor mental health such as depression and anxiety (Oh et al., 2022; Sanborn et al., 2024). These indicators of distress are, in turn, consistently linked to food insecurity across diverse populations (Allen et al., 2018; Arenas et al., 2019; Brown et al., 2025; Sharpe et al., 2016). Beyond the general student population, food insufficiency demonstrates a strong correlation with poor mental health, including higher depressive symptom scores, particularly among low-income women and mothers, with the most severe impact observed in very poor African American mothers (Austin and Smith, 2017; Bailey et al., 2023). Physical factors such as sedentary life (Phillips et al., 2024); Environmental factors, such as noise pollution or cohabiting rooms (Lu et al., 2024), and lifestyle choices, such as sleep regularity (Fischer et al., 2021), frequent consumption of ultra processed food (Duquenne et al., 2024), and stimulants (Boehm et al., 2016), significantly impact sleep. Addressing these needs holistically may work toward reducing dropout rates and promoting better academic achievement for students. Nevertheless, the present findings contribute to the existing academic literature by underscoring the significant interrelationship between food insecurity, sleep duration (Alhasan et al., 2023) and BMI among urban college minority students. These findings highlight severe sleep disparities across different racial and ethnic groups, indicating that systemic or sociocultural factors may significantly impede adequate sleep in these populations.

## 7 Conclusion

This study confirms the strong association between food insecurity and college students, particularly within minorities in a diverse, urban setting in the Bronx, NYC, where an alarmingly high prevalence of 52.1% of participants reported being food insecure. This high rate highlights that food insecurity remains a significant and pressing challenge for students in this demographic 4 years post-pandemic. The evidence shows the complex connections between sleep health, body weight, and food insecurity among minority students, especially Black non-Hispanic individuals in urban colleges. The research strongly supported the existing literature by finding a clear inverse relationship between household income and food insecurity (*p* < 0.0001), emphasizing that financial constraints are a major determinant, though income was not linked to sleep duration. This suggests that stability and resources associated with food security positively contribute to better sleep outcomes. A novel and key contribution is the statistically significant association between inadequate sleep and food insecurity (*p* = 0.007), which suggests a complex, interconnected relationship between these two health determinants. Specifically, the logistic regression analysis demonstrated that students who obtained more sleep were 2.3 times more likely to be food secure *[p* = 0.005, Exp(B)=2.327], further solidifying this relationship. Furthermore, the findings showed a significant association between food insecurity and higher BMI (*p* = 0.0037). Significant disparities in sleep duration related to racial/ethnic status were also noted among the food-insecure subgroup, echoing existing literature on minority student health. Although the correlations between these factors and the GPA were weak, the findings underscore the critical need for comprehensive, multi-level interventions that holistically address food access, sleep health, and economic stability to improve their overall well-being and academic success.

## 8 Recommendations

Addressing the intersectional disadvantages impacting the well-being and academic success of minority students in urban settings necessitates targeted, holistic interventions that recognize the observed association between sleep quality and food security. Institutions must implement culturally informed, comprehensive support by treating poor sleep and food insecurity as interconnected issues. Practical strategies include offering workshops on stress management, sleep hygiene, and food security, given that students from underrepresented backgrounds often face elevated financial strain, decreased belonging, and familial economic pressure, particularly during their first year. For instance, implementing an evidence-based online sleep intervention program—to equip students with cognitive-behavioral tools to achieve restorative sleep—might represents a strategy for mitigating sleep disparities and reframing rest as a critical component of academic resilience. Policymakers should integrate sleep duration metrics into public health surveillance and urban planning frameworks, particularly within high-density corridors like the South Bronx, to systematically mitigate the environmental noise and chronic allostatic load that drive health disparities in marginalized communities. To streamline support and maximize accessibility, college food pantries should be transformed into integrated wellness hubs. This centralized model would extend beyond immediate food and housing aid to consolidate essential services, including nutrition guidance, sleep education, professional counseling, and mental health resources. Policymakers should authorize state and federal grants to fund 'Basic Needs Hubs' that institutionalize student stability by integrating automated SNAP enrollment and emergency meal-sharing directly into university infrastructure. By coordinating these sensitive services, institutions can effectively address students' basic needs and holistic well-being, thereby significantly enhancing academic retention and success.

## 9 Limitations and further investigations

This cross-sectional study presents inherent design limitations. First, the research relied exclusively on subjective, self-reported measures for the key variables of food insecurity, sleep duration, BMI, GPA, and income. No metadata or additional meta-analyses were used to validate these factors. Self-reported health outcomes are vulnerable to systematic selection and information biases, potentially undermining validity. Although established measures were used, our findings were limited by reliance on subjective sleep data and the use of a partial PSQI instrument. Enhanced reliability necessitates administering the full PSQI and integrating objective sleep tracking technology.

Second, the recall bias is an important limitation. Participants must remember their food security status from the previous year, which can lead to inaccurate reporting of their food access and consumption patterns. A similar situation may arise when participants are asked to reflect on their sleep patterns from the previous month. Additionally, the PSQI instrument might be insufficient by itself to account for the unique circumstances faced by college students, who often experience anxiety and poor sleep quality. Moreover, this study did not account for variables such as residential status (e.g., commuting vs. on-campus housing) or access to institutional meal plans, both of which may influence the prevalence of food insecurity. Similarly, the sleep patterns on weekdays vs. weekends, social interactions, study habits, and “digital fatigue” from excessive online classes or video game time were also variables not considered in this study. Furthermore, aspects such as sleep aid, sleep timing, sleep behaviors, and sleep quality require further discussion. Our instrument did not include predictors of poor sleep health. In addition, the relationship between sleep health and working status (students) was not assessed, and its wider implications warrant further attention. Other sleep disruptors, such as environmental factors and the frequency of stimulant, use of cannabis, or energy drink use, were not addressed, although they also affect sleep. Importantly, relying on BMI alone may fail to recognize the ‘allostatic load' that BIPOC students in the South Bronx experience. In this context, a high BMI and poor sleep could be symptoms of chronic socioeconomic stress and systemic challenges, rather than being solely the result of dietary or lifestyle choices.

Third, the generalizability of these findings is limited by the specific sample composition (primarily 86% minority students from two South Bronx, NY institutions) and the exclusive reliance on a quantitative approach. Furthermore, the study's scope was constrained by the omission of key demographic variables such as pregnancy and the experiences of new parents, which may have confounded analyses of gender differences in sleep quality. While unmeasured, the environmental context of “food/sleep deserts” surrounding the institutions presents an important, unaddressed factor. Finally, the instrument's failure to capture contextual sleep variations pertinent to international students further warrants future investigation. Adhering to reporting standards (CHERRIES), we acknowledge potential technical variability inherent in the SurveyMonkey platform. Specifically, device-dependent rendering (e.g., screen size and font display) may have influenced participant engagement. Although ‘view rates' could not be tracked, participation rates were recorded, and duplicate responses were strictly prohibited by the system.

Despite certain limitations, our findings provide valuable insights for minority communities within the collegiate environment. Future initiatives should expand upon these recommendations to further investigate the relationships between sleep insecurity, food insecurity, anxiety, academic performance, and gender. Employing a mixed-methods approach and utilizing probabilistic sampling techniques will enhance representativeness and deepen our understanding of individuals lived experiences. Additionally, a crucial area for further exploration is the impact of excessive technology use prior to bedtime on sleep onset among this student population.

## Data Availability

The raw data supporting the conclusions of this article will be made available by the authors, without undue reservation.

## References

[B1] Abdullaa N. K. Obaida R. R. Qureshia M. N. Asraitia A. A. Janahia M. A. Abu-Qiyasa S. J. . (2023). Relationship between hedonic hunger and subjectively assessed sleep quality and perceived stress among university students: a cross-sectional study. Heliyon 9:e14987. doi: 10.1016/j.heliyon.2023.e14987 37089280 PMC10114148

[B2] Adamovic E. Newton P. House V. (2022). Food insecurity on a college campus: prevalence, determinants, and solutions. J. Am. Coll. Health 70, 58–64. doi: 10.1080/07448481.2020.1725019 32149582

[B3] Al Salmani A. A. Al Shidhani A. Al Qassabi S. S. Al Yaaribi S. A. Al Musharfi A. M. (2020). Prevalence of sleep disorders among university students and its impact on academic performance. Int. J. Adolesc. Youth 25, 974–981. doi: 10.1080/02673843.2020.1815550

[B4] Alafif N. Alruwaili N. W. (2023). Sleep duration, Body Mass Index, and dietary behaviour among KSU students. Nutrients 15:510. doi: 10.3390/nu15030510 36771217 PMC9918940

[B5] Alhasan D. M. Riley N. M. Jackson II W. B. Jackson C. L. (2023). Food insecurity and sleep health by race/ethnicity in the United States. J. Nutr. Sci. 12:e59. doi: 10.1017/jns.2023.18 37252683 PMC10214135

[B6] Al-Khalil Z. Attarian H. Dunietz G. L. Gavidia-Romero R. Knutson K. Johnson D. A. (2024). Sleep health inequities in vulnerable populations: beyond sleep deserts. Sleep Med. 7:100110. doi: 10.1016/j.sleepx.2024.100110 38623559 PMC11017343

[B7] Allen J. (2023). The indirect effects of food insecurity on obesogenic environments. Front. Public Health 10:1052957. doi: 10.3389/fpubh.2022.1052957 36685001 PMC9853452

[B8] Allen N. L. Becerra B. J. Becerra M. B. (2018). Associations between food insecurity and the severity of psychological distress among African-Americans. Ethn. Health 23, 511–520. doi: 10.1080/13557858.2017.1280139 28140616

[B9] Ampofo J. Sun B. Bentum-Micah G. Qinggong L. Changfeng W. Guoan L. . (2025). Investigating the impact of sleep quality on cognitive functions among students in Tokyo, Japan, and London, UK. Front. Sleep 4:1537997. doi: 10.3389/frsle.2025.1537997 41425174 PMC12713871

[B10] Andersen M. L. Alvarenga T. A. Fonseca L. H. Tufik S. (2025). Interactions between co-sleeping, sleep quality, and relationship health: the benefits and challenges of shared sleep environments. J. Health Psychol. 30, 4550–4563. doi: 10.1177/13591053251356355 40772335

[B11] Arenas D. J. Thomas A. Wang J. DeLisser H. M. (2019). A systematic review and meta-analysis of depression, anxiety, and sleep disorders in US adults with food insecurity. J. Gen. Intern. Med. 34, 2874–2882. doi: 10.1007/s11606-019-05202-4 31385212 PMC6854208

[B12] Arzhang P. Sadeghi N. Fatemeh Harchegani A. Rezaei M. Ghaderi M. Saeed-Yekaninejad M. . (2024). Associations between food insecurity and Sleep Duration, Quality, and Disturbance among older adults from six low- and middle-income countries. J. Nutr. Health Aging 28:100018. doi: 10.1016/j.jnha.2023.100018 38267148 PMC12877760

[B13] Austin A. E. Smith M. V. (2017). Examining material hardship in mothers: associations of diaper need and food insufficiency with maternal depressive symptoms. Health Equity 1, 127–133. doi: 10.1089/heq.2016.0023 29082357 PMC5657130

[B14] Azhar S. Ross A. M. Acharya A. Lerner R. Tripathi S. Keller E. . (2024). The little I receive is not enough: a qualitative study of food insecurity in the South Bronx. Food Cult. Soc. 28, 1035–1054. doi: 10.1080/15528014.2024.2406081

[B15] Bailey C. P. Vyas A. Schrum J. Napolitano M. A. (2023). Food insecurity among pregnant and recently pregnant emerging and young adults: an online cross-sectional survey study. J. Hunger Environ. Nutr. 19, 1028–1041. doi: 10.1080/19320248.2023.2261879

[B16] Becerra M. B. Bol B. S. Granados R. Hassija C. (2020). Sleepless in school: the role of social determinants of sleep health among college students. J. Am. Coll. Health 68, 185–191. doi: 10.1080/07448481.2018.1538148 30489219

[B17] Becker S. P. Jarrett M. A. Luebbe A. M. Garner A. A. Burns G. L. Kofler M. J. (2018). Sleep in a large, multi-university sample of college students: sleep problem prevalence, sex differences, and mental health correlates. Sleep Health 4, 174–181. doi: 10.1016/j.sleh.2018.01.001 29555131 PMC5863586

[B18] Bermudez V. N. Fearon-Drake D. Wheelis M. Cohenour M. Suntai Z. Scullin M. K. (2022). Sleep disparities in the first month of college: implications for academic achievement. Sleep Adv. 3:zpac041. doi: 10.1093/sleepadvances/zpac041 37193411 PMC10104382

[B19] Bermúdez-Millán A. Pérez-Escamilla R. Segura-Pérez S. Damio G. Chhabra J. Osborn C. Y. . (2016). Psychological distress mediates the association between food insecurity and suboptimal sleep quality in Latinos with Type 2 Diabetes Mellitus. J. Nutr. 146, 2051–2057. doi: 10.3945/jn.116.231365 27489004 PMC5037870

[B20] Beroukhim G. Esencan E. Seifer D. B. (2022). Impact of sleep patterns upon female neuroendocrinology and reproductive outcomes: a comprehensive review. Reprod. Biol. Endocrinol. 20:16. doi: 10.1186/s12958-022-00889-3 35042515 PMC8764829

[B21] Betancourt-Núñez A. Díaz R. Nava-Amante P. A. Bernal-Orozco M. F. Díaz-López A. González Palacios A. . (2024). Beyond the classroom: the influence of food insecurity, mental health, and sleep quality on university students' academic performance. Foods 13:2508. doi: 10.3390/foods13162508 39200435 PMC11353649

[B22] Boehm M. A. Lei Q. M. Lloyd R. M. Prichard J. R. (2016). Depression, anxiety, and tobacco use: overlapping impediments to sleep in a national sample of college students. J. Am. Coll. Health 64, 565–574. doi: 10.1080/07448481.2016.1205073 27347758

[B23] Broussard J. L. Klein S. (2022). Insufficient sleep and obesity: cause or consequence. Obesity 30, 1914–1916. doi: 10.1002/oby.23539 36042009 PMC9509457

[B24] Brown C. M. Nwakeze P. C. Puri A. Sanchez C. Callender L. Williams E. V. . (2025). Association between food insecurity and mental health among college students in the Bronx, New York (NY). Nutrients 17:3485. doi: 10.3390/nu17213485 41228557 PMC12609369

[B25] Bruening M. Argo K. Payne-Sturges D. Laska M. N. (2017). The struggle is real: a systematic review of food insecurity on postsecondary education campuses. J. Acad. Nutr. Diet. 117, 1767–1791. doi: 10.1016/j.jand.2017.05.022 28754200 PMC6901286

[B26] Bruening M. Brennhofer S. van Woerden I. Todd M. Laska M. (2016). Factors related to the high rates of food insecurity among diverse, urban college freshmen. J. Acad. Nutr. Diet. 116, 1450–1457. doi: 10.1016/j.jand.2016.04.004 27212147 PMC5520984

[B27] Bruening M. van Woerden I. Todd M. Laska M. N. (2018). Hungry to learn: the prevalence and effects of food insecurity on health behaviors and outcomes over time among a diverse sample of university freshmen. Int. J. Behav. Nutr. Phys. Act. 15:9. doi: 10.1186/s12966-018-0647-7 29347963 PMC5774124

[B28] Buysse D. J. Reynolds C. F. Charles F. Monk T. H. Berman S. R. Kupfer D. J. (1989). The Pittsburgh sleep quality index: a new instrument for psychiatric practice and research. Psychiatry Res. 28, 193–213. doi: 10.1016/0165-1781(89)90047-4 2748771

[B29] Caraballo C. Mahajan S. Valero-Elizondo J. Massey D. Lu Y. Roy B. . (2022). Evaluation of temporal trends in racial and ethnic disparities in sleep duration among US adults, 2004-2018. JAMA Netw. Open 5:e226385. doi: 10.1001/jamanetworkopen.2022.6385 35389500 PMC8990329

[B30] Chatterjee D. Hinojosa J. (2023). The Big Apple's bitter bite: food hardship in NYC. The Unheard Third 2022. New Yorkers at the crossroad: challenges and opportunities. Community Service Society Report Finds. Available online at: https://www.cssny.org/news/entry/food-insecurity-is-a-persistent-and-pervasive-problem-in-new-york-city-new- (Accessed October 24, 2025).

[B31] Chen X.-L. Li J. Sun S.-N. Zhang X.-J. Chen J.-H. Wang L.-J. . (2024). Validation of intrinsic capacity and healthy sleep pattern in middle-aged and older adults: a longitudinal Chinese study assessing healthy ageing. J. Nutr. Health Aging 28:100365. doi: 10.1016/j.jnha.2024.100365 39307073 PMC12879214

[B32] Cheng E. R. Luo M. Perkins M. Blake-Lamb T. Kotelchuck M. Arauz-Boudreau A. . (2023). Household food insecurity is associated with obesogenic health behaviors among a low-income cohort of pregnant women in Boston, MA. Public Health Nutr. 26, 943–951. doi: 10.1017/S1368980022000714 35321774 PMC9508288

[B33] Chiang Y. C. Arendt S. Sapp S. (2020). Academic performance, employment, and sleep health: a comparison between working and nonworking students. Int. J. High. Educ. 9, 202–213. doi: 10.5430/ijhe.v9n3p202

[B34] CUNY School of Public Health and Health Policy (2020). The state of food security at CUNY. Healthy CUNY and the Hope Center for College Community and Justice. Available online at: https://cunyurbanfoodpolicy.org/wp-content/uploads/2022/04/CUNY-UFPI_food-security_v07_Final.pdf (Accessed October 24, 2025).

[B35] Darling K. E. Fahrenkamp A. J. Wilson S. M. D'Auria A. L. Sato A. F. (2017). Physical and mental health outcomes associated with prior food insecurity among young adults. J. Health Psychol. 22, 572–581. doi: 10.1177/1359105315609087 26464054

[B36] Degasperi G. Meneo D. Curati S. Cardi V. Baglioni C. Cellini N. (2024). Sleep quality in eating disorders: a systematic review and meta-analysis. Sleep Med. Rev. 77:101969. doi: 10.1016/j.smrv.2024.101969 38959584

[B37] Degenhard S. M. Farmer N. Yang L. Barb J. J. Maki K. A. Wallen G. R. (2025). Specific nutrients mediate the association of food insecurity and Sleep Regularity Index (SRI) in U.S. Adults: NHANES 2011–2014. Nutrients 17:340. doi: 10.3390/nu17020340 39861470 PMC11767890

[B38] Dickinson M. (2023). Skipping meals on campus: college student food insecurity and urban mobility. Food Cult. Soc. 27, 828–845. doi: 10.1080/15528014.2023.2187579

[B39] Dietch J. R. Taylor D. J. Sethi K. Kelly K. Bramoweth A. D. Roane B. M. (2016). Psychometric evaluation of the PSQI in U.S. college students. J. Clin. Sleep Med. 12, 1121–1129. doi: 10.5664/jcsm.6050 27166299 PMC4957190

[B40] Ding M. Keiley M. K. Garza K. B. Duffy P. A. Zizza C. A. (2015). Food insecurity is associated with poor sleep outcomes among US adults. J. Nutr. 145, 615–621. doi: 10.3945/jn.114.199919 25733479

[B41] Dong L. D'Amico E. J. Dickerson D. L. Brown R. A. Palimaru A. I. Johnson C. I. . (2023). Food insecurity, sleep, and cardiometabolic risks in urban American Indian/Alaska Native youth. Sleep Health 9, 4–10. doi: 10.1016/j.sleh.2022.10.003 36328921 PMC9991968

[B42] Dunietz G. L. Braley T. J. Jansen E. C. (2022). Sleep insecurity as a health disparity. J. Clin. Sleep Med. 18:2521. doi: 10.5664/jcsm.10172 35866223 PMC9516577

[B43] Duquenne P. Capperella J. Fezeu L. K. Srour B. Benasi G. Hercberg S. . (2024). The association between ultra-processed food consumption and chronic insomnia in the NutriNet-Santé study. J. Acad. Nutr. Diet. 124, 1109–1117. doi: 10.1016/j.jand.2024.02.015 38423510 PMC12179430

[B44] El Zein A. Shelnutt K. P. Colby S. Vilaro M. J. Zhou W. Greene G. . (2019). Prevalence and correlates of food insecurity among U.S. college students: a multi-institutional study. BMC Public Health 19:660. doi: 10.1186/s12889-019-6943-6 31142305 PMC6542079

[B45] Figorilli M. Velluzzi F. Redolfi S. (2025). Obesity and sleep disorders: a bidirectional relationship. Nutr. Metab. Cardiovasc. Dis. 35:104014. doi: 10.1016/j.numecd.2025.104014 40180826

[B46] Fischer D. Klerman E. B. Phillips A. J. K. (2021). Measuring sleep regularity: theoretical properties and practical usage of existing metrics. Sleep 44:zsab103. doi: 10.1093/sleep/zsab103 33864369 PMC8503839

[B47] Fortin K. Harvey S. Swearingen White S. (2020). Hidden hunger: understanding the complexity of food insecurity among college students. J. Am. Coll. Nutr. 40, 242–252. doi: 10.1080/07315724.2020.1754304 33048013

[B48] Frosztega W. Sroczyński M. Michałek M. Wieckiewicz M. Madziarska K. Martynowicz H. (2025). Gallstones, obesity, insulin resistance, and obstructive sleep apnea - current knowledge on the topic: a literature review. Dent. Med. Probl. 62, 987–992. doi: 10.17219/dmp/204402 41247085

[B49] Gaultney J. F. (2010). The prevalence of sleep disorders in college students: impact on academic performance. J. Am. Coll. Health 59, 91–97. doi: 10.1080/07448481.2010.483708 20864434

[B50] Goldrick-Rab S. Baker-Smith C. Coca V. Looker E. Williams T. (2019). College and University basic needs insecurity: A national #realcollege survey report. The Hope Center. Available online at: https://hope.temple.edu/sites/hope/files/media/document/HOPE_realcollege_National_report_digital.pdf (Accessed November 14, 2025).

[B51] Goldstein S. J. Gaston S. A. McGrath J. A. Jackson C. L. (2020). Sleep health and serious psychological distress: a nationally representative study of the United States among White, Black, and Hispanic/Latinx adults. Nat. Sci. Sleep 12, 1091–1104. doi: 10.2147/NSS.S268087 33299371 PMC7721291

[B52] Guerithault N. McClure S. M. Ojinnaka C. O. Braden B. B. Bruening M. (2022). Resting-state functional connectivity differences in college students with and without food insecurity. Nutrients 14:2064. doi: 10.3390/nu14102064 35631206 PMC9145508

[B53] Hagedorn R. L. Olfert M. D. MacNell L. Houghtaling B. Hood L. B. Savoie Roskos M. R. . (2021). College student sleep quality and mental and physical health are associated with food insecurity in a multi-campus study. Public Health Nutr. 24, 4305–4312. doi: 10.1017/S1368980021001191 33745495 PMC8385605

[B54] Hale L. Do D. P. (2007). Racial differences in self-reports of sleep duration in a population-based study. Sleep 30, 1096–1103. doi: 10.1093/sleep/30.9.1096 17910381 PMC1978399

[B55] Hales L. J. (2024). Food insecurity in U.S. households varies across race and ethnicity. Household food insecurity across race and ethnicity in the United States, 2016–21. USDA, Economic Research Service. Available online at: https://www.ers.usda.gov/data-products/charts-of-note/chart-detail?chartId=108925 (Accessed November 14, 2025).

[B56] Hamilton W. L. Cook J. T. Thompson W. W. Buron L. F. Frongillo E. A. Olson C. M. . (1997). Household food security in the United States in 1995. Summary Report of the Food Security Measurement Project. Prepared for U.S. Department of Agriculture, Food and Consumer Service. Available online at: https://fns-prod.azureedge.us/sites/default/files/TECH_RPT.PDF (Accessed September 12, 2025).

[B57] Hanson M. (2025). College enrollment and student demographic statistics. Educational Data Initiative. Available online at: https://educationdata.org/college-enrollment-statistics (Accessed November 14, 2025).

[B58] Hershner S. D. Chervin R. D. (2014). Causes and consequences of sleepiness among college students. Nat. Sci. Sleep 6, 73–84. doi: 10.2147/NSS.S62907 25018659 PMC4075951

[B59] Hill V. M. Rebar A. L. Ferguson S. A. Shriane A. E. Vincent G. E. (2022). Go to bed! A systematic review and meta-analysis of bedtime procrastination correlates and sleep outcomes. Sleep Med. Rev. 66:101697. doi: 10.1016/j.smrv.2022.101697 36375334

[B60] Hirshkowitz M. Whiton K. Albert S. M. Alessi C. Bruni O. DonCarlos L. . (2015). National Sleep Foundation's sleep time duration recommendations: methodology and results summary. Sleep Health 1, 40–43. doi: 10.1016/j.sleh.2014.12.010 29073412

[B61] Hung C. T. Wu S. E. Chen Y. H. Soong C. Y. Chiang C. P. Wang W. M. (2024). The evaluation of synchronous and asynchronous online learning: student experience, learning outcomes, and cognitive load. BMC Med. Educ. 24:326. doi: 10.1186/s12909-024-05311-7 38519950 PMC10960437

[B62] Hussain B. M. Ryan R. Deierlein A. L. Lal S. Bihuniak J. D. Parekh N. (2022). Food insecurity and health behaviors among a sample of undergraduate students at an urban university. J. Hunger Environ. Nutr. 18, 65–80. doi: 10.1080/19320248.2022.2119119

[B63] IBM Corp (2025). IBM SPSS Statistics for Windows (Version 29.0). [Computer Software]. Armonk, NY: IBM Corp.

[B64] Jackson C. L. Powell-Wiley T. M. Gaston S. A. Andrews M. R. Tamura K. Ramos A. (2020a). Racial/ethnic disparities in sleep health and potential interventions among women in the United States. J. Womens Health 29, 435–442. doi: 10.1089/jwh.2020.8329 32096683 PMC7097680

[B65] Jackson C. L. Walker J. R. Brown M. K. Das R. Jones N. L. (2020b). A workshop report on the causes and consequences of sleep health disparities. Sleep 43:zsaa037. doi: 10.1093/sleep/zsaa037 32154560 PMC7420527

[B66] Jacob L. Smith L. Kostev K. Oh H. Gyasi R. M. López Sánchez G. F. . (2023). Food insecurity and insomnia-related symptoms among adults from low- and middle-income countries. J. Sleep Res. 32:e13852. doi: 10.1111/jsr.13852 36808652

[B67] Johnson D. A. Billings M. E. Hale L. (2018). Environmental determinants of insufficient sleep and sleep disorders: implications for population health. Curr. Epidemiol. Rep. 5, 61–69. doi: 10.1007/s40471-018-0139-y 29984131 PMC6033330

[B68] Johnson D. A. Jackson C. L. Williams N. J. Alcántara C. (2019). Are sleep patterns influenced by race/ethnicity - a marker of relative advantage or disadvantage? Evidence to date. Nat. Sci. Sleep 11, 79–95. doi: 10.2147/NSS.S169312 31440109 PMC6664254

[B69] Jordan M. L. Perez-Escamilla R. Desai M. M. Shamah-Levy T. (2016). Household food insecurity and sleep patterns among Mexican adults: results from ENSANUT-2012. J. Immigr. Minor. Health 18, 1093–1103. doi: 10.1007/s10903-015-0246-5 26163336

[B70] Kendrick A. Fantasia H. C. Morse B. Willis D. E. (2022). Food insecurity in college students: a concept analysis. Nurs. Forum 57, 898–904. doi: 10.1111/nuf.12737 35616363

[B71] Kim Y. Ramos A. R. Carver C. S. Ting A. Hahn K. Mossavar-Rahmani Y. . (2021). Marital status and gender associated with sleep health among Hispanics/Latinos in the US: results from HCHS/SOL and Sueño Ancillary Studies. Behav. Sleep Med. 20, 531–542. doi: 10.1080/15402002.2021.1953499 34308745 PMC8784567

[B72] Knol L. L. Robb C. A. McKinley E. M. Wood M. (2017). Food insecurity, self-rated health, and obesity among college students. Am. J. Health Educ. 48, 248–255. doi: 10.1080/19325037.2017.1316689

[B73] Kopels M. C. Shattuck E. C. Rocha J. Roulette C. J. (2024). Investigating the linkages between food insecurity, psychological distress, and poor sleep outcomes among U.S. college students. Am. J. Hum. Biol. 36:e24032. doi: 10.1002/ajhb.24032 38116753

[B74] Lajunen T. Gaygisiz E. Wang W. (2023). Sleep and happiness: socio-economic, population and cultural correlates of sleep duration and subjective well-being in 52 countries. Front. Sleep 2:1118384. doi: 10.3389/frsle.2023.1118384 41426472 PMC12713995

[B75] Landry M. J. Heying E. Qamar Z. Hagedorn-Hatfield R. L. Savoie-Roskos M. R. Cuite C. L. . (2024). Advancing college food security: priority research gaps. Nutr. Res. Rev. 37, 108–120. doi: 10.1017/S0954422423000094 37158045

[B76] Lastella M. Miller D. J. Montero A. Sprajcer M. Ferguson S. A. Browne M. . (2025). Sleep on it: a pilot study exploring the impact of sexual activity on sleep outcomes in cohabiting couples. Sleep Health 11, 198–205. doi: 10.1016/j.sleh.2024.11.004 40016080

[B77] Lastella M. Rigney G. Browne M. Sargent C. (2020). Electronic device use in bed reduces sleep duration and quality in adults. Sleep Biol. Rhythms 18, 121–129. doi: 10.1007/s41105-019-00251-y

[B78] Leckrone B. (2025). Full-Time vs. Part-Time Student: What's the Difference? Best Colleges. Available online at: https://www.bestcolleges.com/blog/full-time-vs-part-time-student/ (Accessed November 14, 2025).

[B79] Lee S. Deason K. Rancourt D. Gray H. L. (2021). Disentangling the relationship between food insecurity and poor sleep health. Ecol. Food Nutr. 60, 580–595. doi: 10.1080/03670244.2021.1926245 34032535

[B80] Li Q. (2021). The association between sleep duration and excess body weight of the American adult population: a cross-sectional study of the national health and nutrition examination survey 2015–2016. BMC Public Health 21:335. doi: 10.1186/s12889-021-10369-9 33573618 PMC7879643

[B81] Liu Y. Njai R. S. Greenlund K. J. Chapman D. P. Croft J. B. (2014). Relationships between housing and food insecurity, frequent mental distress, and insufficient sleep among adults in 12 US states, 2009. Prev. Chronic Dis. 11:130334. doi: 10.5888/pcd11.130334 24625361 PMC3958143

[B82] Lu S. Stone J. E. Klerman E. B. McHill A. W. Barger L. K. Robbins R. . (2024). The organization of sleep–wake patterns around daily schedules in college students. Sleep 47:zsad278. doi: 10.1093/sleep/zsad278 37930792 PMC11381563

[B83] Ma C. Ho S. K. M. Singh S. Choi M. Y. (2021). Gender disparities in food security, dietary intake, and nutritional health in the United States. Am. J. Gastroenterol. 116, 584–592. doi: 10.14309/ajg.0000000000001118 33443848

[B84] Martinez-Miller E. E. Gaston S. A. Jackson II W. B. Jackson C. L. (2025). Sleep health by sexual orientation among women in the United States and interrelations with race/ethnicity, age, and generational cohort. Int. J. Womens Health 17, 1563–1586. doi: 10.2147/IJWH.S485205 40463626 PMC12132062

[B85] Mazloomi S. N. Talebi S. Kazemi M. Ghoreishy S. M. Moosavian S. P. Amirian P. . (2023). Food insecurity is associated with the sleep quality and quantity in adults: a systematic review and meta-analysis. Public Health Nutr. 26, 792–802. doi: 10.1017/S1368980022002488 36416108 PMC10131157

[B86] Mbous Y. P. V. Nili M. Mohamed R. Dwibedi N. (2022). Psychosocial correlates of insomnia among college students. Prev. Chronic Dis. 19:E60. doi: 10.5888/pcd19.220060 36108290 PMC9480843

[B87] Meira e Cruz M. Andersen M. L. (2025). Sleep, sex and psychosocial health: expanding the horizons of behavioral sleep medicine. Dent. Med. Probl. 62, 771–773. doi: 10.17219/dmp/209574 41099537

[B88] Meza A. Altman E. Martinez S. Leung C. W. (2019). “It's a feeling that one is not worth food”: a qualitative study exploring the psychosocial experience and academic consequences of food insecurity among college students. J. Acad. Nutr. Diet. 119, 1713–1721. doi: 10.1016/j.jand.2018.09.006 30553586 PMC6561835

[B89] Mitchell A. Ellison B. Bruening M. (2022). Persistent and episodic food insecurity and associated coping strategies among college students. J. Nutr. Educ. Behav. 54, 972–981. doi: 10.1016/j.jneb.2022.06.003 36184354

[B90] Nagata J. M. Palar K. Gooding H. C. Garber A. K. Whittle H. J. Bibbins-Domingo K. . (2019). Food insecurity is associated with poorer mental health and sleep outcomes in young adults HHS public access. J. Adolesc. Health 65, 805–811. doi: 10.1016/j.jadohealth.2019.08.010 31587956 PMC6874757

[B91] National Sleep Foundation (2016). Available online at: https://www.thensf.org/nsf-cta-ansi-standards/ (Accessed September 13, 2025).

[B92] New York State Department of Health (2023). Self-reported food insecurity among New York State adults, by county. Information for Action # 2023-12. Available online at: https://www.health.ny.gov/statistics/prevention/injury_prevention/information_for_action/docs/2023-12_ifa_report.pdf (Accessed September 12, 2025)

[B93] Nikolaus C. J. An R. Ellison B. Nickols-Richardson S. M. (2020). Food insecurity among college students in the United States: a scoping review. Adv. Nutr. 11, 327–348. doi: 10.1093/advances/nmz111 31644787 PMC7442331

[B94] Nyer M. Farabaugh A. Fehling K. Soskin D. Holt D. Papakostas G. I. . (2013). Relationship between sleep disturbance and depression, anxiety, and functioning in college students. Depress. Anxiety 30, 873–880. doi: 10.1002/da.22064 23681944 PMC3791314

[B95] Oh H. Smith L. Jacob L. Du J. Shin J. I. Zhou S. . (2022). Food insecurity and mental health among young adult college students in the United States. J. Affect. Disord. 303, 359–363. doi: 10.1016/j.jad.2022.02.009 35157947

[B96] Osei Bonsu E. Afetor M. Munkaila L. Okwei R. Nachibi S. U. Adjei B. N. . (2023). Association of food insecurity and sleep difficulty among 189,619 school-going adolescents: a study from the global in-school students survey. Front. Public Health 11:1212254. doi: 10.3389/fpubh.2023.1212254 37501946 PMC10369053

[B97] Patrick M. E. Griffin J. Huntley E. D. Maggs J. L. (2016). Energy drinks and binge drinking predict college students' sleep quantity, quality, and tiredness. Behav. Sleep Med. 16, 92–105. doi: 10.1080/15402002.2016.1173554 27183506 PMC5435568

[B98] Patton-López M. M. López-Cevallos D. F. Cancel-Tirado D. I. Vazquez L. (2014). Prevalence and correlates of food insecurity among students attending a midsize rural university in Oregon. J. Nutr. Educ. Behav. 46, 209–214. doi: 10.1016/j.jneb.2013.10.007 24406268

[B99] Phillips H. Gaiser J. Hubert P. Duffy V. (2024). Cross-sectional results: support need to address sleep quality in college students through healthy diet and physical activity. J. Nutr. Educ. Behav. 56, S80–S81. doi: 10.1016/j.jneb.2024.05.183

[B100] Pilcher J. J. Rummel E. G. Lamm C. (2024). Sleep quality, sleep quantity, and sleep timing: contrasts in Austrian and U.S. college students. Front. Sleep 3:1487739. doi: 10.3389/frsle.2024.1487739 41424489 PMC12713796

[B101] Porras-Pérez E. Romero-Cabrera J. L. Díaz-Cáceres A. Serrán-Jiménez A. Arenas-Montes J. Peña-Orihuela P. J. . (2025). Food insecurity and its cardiovascular implications in underresourced communities. J. Am. Heart Assoc. 14:037457. doi: 10.1161/JAHA.124.037457 40082777 PMC12132623

[B102] Powers A. (2024). SNAP acceptance and food insecurity at the Brunswick-Topsham Land Trust Farmers' Market (Thesis). University of Maine, Orono, ME. Available online at: https://digitalcommons.library.umaine.edu/honors/881 (Accessed November 7, 2025).

[B103] Rabbitt M. P. Hales L. J. Burke M. P. Coleman-Jensen A. (2023). Household Food Security in the United States in 2022. (Report No. ERR-325), U.S. Department of Agriculture, Economic Research Service. Available online at: https://www.ers.usda.gov/webdocs/publications/107703/err-325.pdf?v=7814.4 (Accessed November 7, 2025) doi: 10.32747/2023.8134351.ers

[B104] Radtke M. D. Steinberg F. M. Scherr R. E. (2024). Methods for assessing health outcomes associated with food insecurity in the United States college student population: a narrative review. Adv. Nutr. 15:100131. doi: 10.1016/j.advnut.2023.10.004 37865221 PMC10831897

[B105] Rizk R. Haddad C. Sacre H. Malaeb D. Wachten H. Strahler J. . (2023). Assessing the relationship between food insecurity and lifestyle behaviors among university students: a comparative study between Lebanon and Germany. BMC Public Health 23:807. doi: 10.1186/s12889-023-15694-9 37138254 PMC10154760

[B106] Robbins R. Quan S. F. Buysse D. J. Weaver M. D. Walker M. P. Drake C. L. . (2022). A nationally representative survey assessing restorative sleep in US adults. Front. Sleep 1:935228. doi: 10.3389/frsle.2022.935228 36042946 PMC9423762

[B107] Ryan R. A. Murphy B. Deierlein A. L. Lal S. Parekh N. Bihuniak J. D. (2022). Food insecurity, associated health behaviors, and academic performance among urban university undergraduate students. J. Nutr. Educ. Behav. 54, 269–275. doi: 10.1016/j.jneb.2021.08.008 34758921

[B108] Sanborn J. Jones H. E. Manze M. Twiste T. Freudenberg N. (2024). The cumulative impact of unmet essential needs on indicators of attrition: findings from a public university population-based sample of students in the Bronx, NY. J. Urban Health 101, 764–774. doi: 10.1007/s11524-024-00872-w 38955896 PMC11329490

[B109] Seicean S. Neuhauser D. Strohl K. Redline S. (2011). An exploration of differences in sleep characteristics between Mexico-born US immigrants and other Americans to address the Hispanic paradox. Sleep 34, 1021–1031. doi: 10.5665/SLEEP.1154 21804664 PMC3138157

[B110] Seweryn P. Orzeszek S. M. Waliszewska-Prosół M. Jenča A. Osiewicz M. Paradowska-Stolarz A. . (2023). Relationship between pain severity, satisfaction with life and the quality of sleep in Polish adults with temporomandibular disorders. Dent. Med. Probl. 60, 609–617. doi: 10.17219/dmp/171894 37873974

[B111] Sharpe P. A. Whitaker K. Alia K. A. Wilcox S. Hutto B. (2016). Dietary intake, behaviors and psychosocial factors among women from food-secure and food-insecure households in the United States. Ethn. Dis. 26, 139–146. doi: 10.18865/ed.26.2.139 27103763 PMC4836893

[B112] Silva M. R. Kleinert W. L. Sheppard A. V. Cantrell K. A. Freeman-Coppadge D. J. Tsoy E. . (2017). The relationship between food security, housing stability, and school performance among college students in an urban university. J. Coll. Stud. Retent. 19, 284–299. doi: 10.1177/1521025115621918

[B113] Singh S. Esaryk E. E. Meza E. Britton T. Martinez S. M. (2024). Disparities in food insecurity and academic achievement among California Public University Students: an intersectional approach. Nutrients 16:3728. doi: 10.3390/nu16213728 39519560 PMC11547758

[B114] Slotnick M. J. Ansari S. Parnarouskis L. Gearhardt A. N. Wolfson J. A. Leung C. W. (2023). Persistent and changing food insecurity among students at a Midwestern University is associated with behavioral and mental health outcomes. Am. J. Health Promot. 38, 483–491. doi: 10.1177/08901171231224102 38130004 PMC11044134

[B115] Taylor L. C. Delavega E. Jin S. W. Neely-Barnes S. L. Elswick S. E. (2019). The prevalence and correlates of food insecurity among students at a multi-campus university. J. Poverty 23, 621–633. doi: 10.1080/10875549.2019.1656141

[B116] Troxel W. M. Haas A. Ghosh-Dastidar B. Richardson A. S. Hale L. Buysse D. J. . (2020). Food insecurity is associated with objectively measured sleep problems. Behav. Sleep Med. 18, 719–729. doi: 10.1080/15402002.2019.1669605 31545653 PMC8152928

[B117] Tseng K. K. Park S. H. Shearston J. A. Lee L. Weitzman M. (2017). Parental psychological distress and family food insecurity: sad dads in hungry homes. J. Dev. Behav. Pediatr. 38, 611–618. doi: 10.1097/DBP.0000000000000481 28742541

[B118] United States Department of Agriculture Economic Research Service (2025). Definitions of food security. Available online at: https://www.ers.usda.gov/topics/food-nutrition-assistance/food-security-in-the-us/definitions-of-food-security (Accessed November 28, 2025).

[B119] US Census Bureau (2025). Historical income tables. Households. Table H-1. Income Limits for Each Fifth and Top 5 Percent. All races. US Department of Commerce. Available online at: https://www.census.gov/data/tables/time-series/demo/income-poverty/historical-income-households.html (Accessed Feburary 20, 2026).

[B120] USDA Economic Research Service (2012). U.S. Household Food Security survey module: Three-stage design with screeners. Available online at: https://www.ers.usda.gov/media/6422/us-household-food-security-survey-module.pdf (Accessed September 12, 2025).

[B121] USDA Food and Nutrition Service (2025). Students. Supplemental Nutritional Assistance Program. Available online at: https://www.fns.usda.gov/snap/students#:~:text=Most%20SNAP%20eligibility%20rules%20apply,of%20higher%20education%20described%20here (Accessed Feburary 20, 2026).

[B122] Van Reen E. Roane B. M. Barker D. H. McGeary J. E. Borsari B. Carskadon M. A. (2016). Current alcohol use is associated with sleep patterns in first-year college students. Sleep 39, 1321–1326. doi: 10.5665/sleep.5862 27070138 PMC4863222

[B123] Vazquez-Colon N. Lopez-Cepero A. Amaya C. Tucker K. L. Kiefe C. I. Person S. D. . (2024). The association between food insecurity and insomnia symptoms among young adults in Puerto Rico and the mediating role of psychological distress symptoms. Int. J. Environ. Res. Public Health 21:1296. doi: 10.3390/ijerph21101296 39457271 PMC11507534

[B124] Villela-Maciel F. Tuchtenhagen-Wendt A. Miranda-Demenech L. Carvalho-Dumith S. (2023). Factors associated with sleep quality in university students. Ciênc. Saúde Colet. 28, 1187–1198. doi: 10.1590/1413-81232023284.14132022 37042899

[B125] Watson N. F. Badr M. S. Belenky G. Bliwise D. L. Buxton O. M. Buysse D. . (2015). Recommended amount of sleep for a healthy adult: a joint consensus statement of the American Academy of Sleep Medicine and Sleep Research Society. J. Clin. Sleep Med. 11, 591–592. doi: 10.5664/jcsm.4758 25979105 PMC4442216

[B126] Wattick R. A. Hagedorn R. L. Olfert M. D. (2018). Relationship between diet and mental health in a young adult Appalachian College population. Nutrients 10:957. doi: 10.3390/nu10080957 30044399 PMC6115820

[B127] Whinnery J. Jackson N. Rattanaumpawan P. Grandner M. A. (2014). Short and long sleep duration associated with race/ethnicity, sociodemographics, and socioeconomic position. Sleep 37, 601–611. doi: 10.5665/sleep.3508 24587584 PMC3920327

[B128] Wieckiewicz M. Winocur E. (2023). Editorial: Orofacial pain, bruxism, and sleep, volume II. Front. Neurol. 14:1331275. doi: 10.3389/fneur.2023.1331275 38357292 PMC10866288

[B129] Xu Q. Lin Z. Chen Y. Huang M. (2025). Association between sleep duration and patterns and obesity: a cross-sectional study of the 2007-2018 national health and nutrition examination survey. BMC Public Health 25:1460. doi: 10.1186/s12889-025-22433-9 40259262 PMC12010617

[B130] Zhou J. Qu J. Ji S. Bu Y. Hu Y. Sun H. . (2022). Research trends in college students' sleep from 2012 to 2021: a bibliometric analysis. Front. Psychiatry 13:1005459. doi: 10.3389/fpsyt.2022.1005459 36203831 PMC9530190

